# Long‐Term Firocoxib Use in Horses

**DOI:** 10.1111/jvim.70117

**Published:** 2025-05-02

**Authors:** Iuri Buzelato Carli, Langdon Fielding

**Affiliations:** ^1^ Loomis Basin Equine Medical Center Penryn California USA

**Keywords:** creatinine, lameness, NSAID, renal, surgery

## Abstract

**Background:**

Many horses receive firocoxib over multiple years, and studies evaluating hematologic and biochemical values in these animals are lacking.

**Objective:**

To describe the hematologic and biochemical values of horses receiving long‐term firocoxib.

**Animals:**

A total of 79 horses receiving long‐term firocoxib and 153 horses not receiving long‐term firocoxib and not presenting for a specific medical problem.

**Methods:**

Retrospective study comparing horses receiving firocoxib (F group) and no firocoxib (NF group). Signalment, hematologic, and biochemical values were compared between the two groups. Simple linear regression was used to evaluate the relationship between the duration of firocoxib administration and specific laboratory variables.

**Results:**

Horses receiving long‐term firocoxib (F) were 19 (5–33) years as compared to 15 (1–33) years for the NF group (*p* < 0.0001). The most common reason for receiving firocoxib was osteoarthritis in 44/79 (56%) of the animals in the F group. Horses in the F group had a total protein concentration of 6.6 (5.8–8.0) g/dL as compared to 6.5 (5.1–8) g/dL in the NF group (*p* = 0.03). The sodium concentration for F horses was 139 (133–143) mmol/L as compared to 138 (129–145) mmol/L for NF horses (*p* = 0.01). Total white blood cell count was 6.11 (2.82–14.36) 10^3^ cells/uL in the F group as compared to 6.46 (3.04–14.71) 10^3^ cells/uL in the NF group (*p* = 0.03). None of the laboratory variables were associated with the duration of firocoxib administration.

**Conclusions and Clinical Importance:**

Many horses receive firocoxib for multiple years, and equine practitioners should be aware that expected changes in laboratory values are minimal.

**Abbreviations**
F: firocoxib
NF: no firocoxib

## 1 Introduction

Firocoxib is a non‐steroidal anti‐inflammatory drug (NSAID) classified as a coxib, a class of drugs that is known for selective inhibition of the COX‐2 enzyme that involves the inflammatory process [1, 2, 3, 4, 5, 6, 7]. Equine practitioners use firocoxib for long‐term treatment in horses for a number of reasons. First, osteoarthritis is common in horses, and firocoxib improves specific variables such as range of motion and pain on palpation as compared to phenylbutazone [2, 8]. Second, when compared to vedaprofen, firocoxib clinically improves more chronically lame horses with osteoarthritis [9]. Third, firocoxib induces fewer gastrointestinal adverse effects when compared to phenylbutazone [10]. Overall, firocoxib provides good absorption, a longer half‐life than other NSAIDs, and presents an acceptable safety profile to equine practitioners [3, 11]. Firocoxib is labeled for oral use for 14 days in horses (Equioxx Package Insert, Boehringer Ingelheim Animal Health USA Inc., Duluth, GA, USA). However, despite its widespread use, the effects of long‐term firocoxib (> 14 days) on biochemical and hematologic values in horses are unknown.

Coxibs are used in other species including dogs and people [12, 13]. In contrast to horses, there are studies in both of these species describing the long‐term effects of coxibs on laboratory variables. In dogs, long‐term use of coxibs is associated with a mild increase in serum creatinine concentration [4]. Other biochemical and hematologic changes associated with long‐term use are not identified [4]. However, other studies have had mixed results with only mild changes in renal values [7, 14]. Studies of long‐term coxib treatment in people reveal a low incidence of gastrointestinal adverse effects [15, 16, 17]. While firocoxib is used long‐term in dogs, horses, and people, equine medicine lacks the research needed to support this common practice.

The objective of this study was to conduct a retrospective evaluation comparing horses receiving long‐term firocoxib with those not receiving long‐term firocoxib and specifically to determine if there are significant changes in laboratory variables that would be clinically relevant. The hypothesis is that long‐term firocoxib treatment would be associated with increases in concentrations of serum creatinine and BUN, a decrease in plasma protein concentration, and a decrease in total white cell count.

## 2 Materials and Methods

Medical records of horses from the Loomis Basin Equine Medical Center from September 1, 2021 to September 30, 2021 were included for initial analysis. A specific wellness protocol was offered during this month for horses enrolling in an annual preventative care program. As part of this enrollment, a complete blood count and chemistry panel was performed in addition to a manual PCV and total protein measured using the microhematocrit method and refractometer. The results were stored in the clinic's medical database and accessed retrospectively for the purposes of the study. Exclusion criteria included any horse that presented to the hospital for medical treatment or for evaluation of a specific medical condition.

Information obtained from the medical records included signalment (age, sex, weight, and breed), firocoxib treatment status (duration of treatment if treated), packed cell volume (PCV), manual total protein concentration (TP), serum albumin concentration, serum globulin concentration, serum creatinine concentration, blood urea nitrogen concentration (BUN), serum sodium concentration, serum potassium concentration, serum chloride concentration, total white blood count (WBC), and neutrophil count. When weight was recorded, the dose of firocoxib was calculated. Breed was categorized as Arabian, Quarter Horse, Paint Horse, Thoroughbred, and other.

Horses receiving firocoxib for 2 or more months were categorized as the F group, and horses not receiving firocoxib were categorized as the NF group. Horses that received firocoxib for less than 2 months were excluded from the study. Duration of firocoxib treatment and dosage was recorded in months based on the first date of purchase and/or prescription of the medication. For the NF group, records were reviewed for history of firocoxib prescriptions from an outside pharmacy or for firocoxib treatment that was discontinued due to adverse effects.

Additionally, the reason for long‐term firocoxib treatment was recorded from the medical record and categorized as osteoarthritis, laminitis, orthopedic soft tissue injury (e.g., tendon or ligament tear), navicular disease, other chronic lameness, and non‐lameness problem. Animals could have more than one diagnosis category for the cause of firocoxib use if indicated by the medical record.

Data were reported as mean ± SD for normally distributed data and median (range) for non‐normally distributed data. Normality was determined using the Kolmogorov–Smirnov test. For continuous variables (e.g., age, weight, laboratory values), the firocoxib (F) and no firocoxib (NF) groups were compared using an unpaired *t*‐test (normally distributed data) or a Mann–Whitney test (non‐normally distributed data). For categorical variables (e.g., sex, breed), the F and NF groups were compared using Fisher's exact test.

For horses in the firocoxib group, simple linear regression was used to assess relationships between the duration of firocoxib treatment (number of months) and specific hematologic and biochemical variables. Laboratory variables with *p* < 0.2 in the simple regression analysis were selected to be included in the multiple linear regression model selection, including sex and age as mandatory explanatory variables. A forward stepwise process was used for model selection when multiple variables were included. A commercially available statistical software package was used for analysis (GraphPad Prism version 10.02 for Windows, GraphPad Software).

## 3 Results

A total of 408 horses were included for initial evaluation. Of these animals, 176 were removed from the study group based on the exclusion criteria. Of the remaining 232 horses, 153/232 (66%) cases were not receiving long‐term firocoxib (NF) and 79/232 (34%) cases were receiving long‐term firocoxib (F). The duration of firocoxib treatment was 10 (2–52) months. Within the group of animals receiving firocoxib, 17/79 (22%) patients were receiving firocoxib for 2–4 months, 28/79 (35%) for 5–11 months, 17/79 (22%) for 12–24 months, and 17/79 (22%) patients were receiving firocoxib for greater than 24 months. There were 60/79 (76%) of horses receiving firocoxib with a recorded body weight of 487 ± 108 kg. These horses received a firocoxib dosage of 0.13 ± 0.08 mg/kg which is similar to the dosage used in other equine studies [18, 19].

The age of the 153 horses not receiving long‐term firocoxib (NF) was 15 (1–33) years and was significantly lower than the 79 horses receiving long‐term firocoxib (F) 19 (5–33) years (*p* < 0.0001). The Arabian breed was more common in the firocoxib group (19/79; 25%) as compared to the non‐firocoxib group (17/153; 11%; *p* = 0.01). No other significant differences in breed distribution were identified between the groups. There were 62/153 (41%) mares in the NF group and 34/79 (43%) mares in the F group, which was not significantly different (*p* = 0.78).

The main reason for horses receiving long‐term firocoxib was osteoarthritis (44/79; 56%), followed by animals where the medical record did not indicate a diagnosis (17/79; 21%), laminitis (8/79; 10%), orthopedic soft tissue injury (8/79; 10%), navicular syndrome (5/79; 6%), chronic lameness (5/79; 6%), and non‐lameness problems (2/79; 2%). Nine of 79 (11%) horses had more than one diagnosis as a reason to receive firocoxib.

Laboratory values for the F and NF groups are reported in Table 1. Horses in the F group had a total protein concentration of 6.6 (5.8–8.0) g/dL as compared to 6.5 (5.1–8.0) g/dL in the NF group (*p* = 0.03). Two of the horses in the NF group had total protein concentrations below the reference range whereas no horses in the F group had concentrations below the normal range. The sodium concentration for F horses was 139 (133–143) mmol/L as compared to 138 (129–145) mmol/L for NF horses (*p* = 0.01). Five horses in the NF group had a sodium concentration above the normal range whereas only one horse in the F group had a concentration above the normal range. Total white blood cell count was 6.11 (2.82–14.36) 10^3^ cells/uL in the F group as compared to 6.46 (3.04–14.71) 10^3^ cells/uL in the NF group (*p* = 0.03). Twelve horses in the NF group had a WBC concentration below the normal range whereas 15 horses in the F group had a WBC concentration below the normal range. There was no difference in BUN and creatinine concentration between the F and NF groups.

**TABLE 1:** Comparison of laboratory values between the F (firocoxib) and NF (no firocoxib) groups.

Variable	Firocoxib (F)	*N*	No firocoxib (NF)	*N*	*p*	Reference range
White blood cells (WBC; 10^3^ cells/μL)	6.11 (2.82–14.36)	79	6.46 (3.04–14.71)	153	0.03	5.00–12.00
Neutrophils (10^3^ cells/uL)	3.7 (1.96–12.11)	79	3.72 (2.01–13.04)	153	0.49	2.18–6.96
Packed cell volume (PCV; %)	34 (24–51)	78	35 (26–57)	153	0.22	30.0–49.0
Total protein (TP) – manual (g/dL)	6.6 (5.8–8.0)	78	6.5 (5.1–8)	153	0.03	5.5–8.0
Globulins (g/dL)	3.7 (2.4–4.8)	76	3.6 (2.4–4.9)	153	0.20	2.8–4.8
Albumin (g/dL)	3 (2.5–3.5)	79	3 (2.4–3.5)	153	0.35	2.8–3.9
Creatinine (mg/dL)	1 (0.5–1.8)	79	1 (0.5–4.2)	153	0.58	0.7–1.8
Blood urea nitrogen (BUN; mg/dL)	18.0 (8.6–28.3)	78	17.9 (9.5–52.1)	151	0.92	10.0–25.0
Sodium (Na; mmol/L)	139 (133–143)	79	138 (129–145)	151	0.02	130–142
Potassium (K; mmol/L)	4.1 (2.6–7.7)	79	4.2 (2.4–7.7)	149	0.16	2.4–4.6
Chloride (Cl; mmol/L)	99 (90–108)	79	99 (87–107)	150	0.78	95–108

*Note:* Variables were compared using a Mann–Whitney test and *p* < 0.05 was considered statistically significant. Data are reported as mean ± SD or median (range).

Simple linear regression analysis between firocoxib duration and each laboratory variable identified four variables (WBC, neutrophils, albumin, and globulin) with *p* < 0.2 (Table 2). Simple linear regression between age (explanatory variable) and each laboratory variable (outcome) identified creatinine (*p* = 0.03) and albumin (*p* = 0.07) with *p* < 0.2. Simple linear regression between sex (explanatory variable) and each laboratory variable (outcome) identified WBC (*p* = 0.07) and BUN (*p* = 0.06) with *p* < 0.2. Based on these results, a multiple linear regression model for albumin with age and firocoxib duration was evaluated (*p* = 0.65) as well as a multiple linear regression model for WBC with sex and duration of firocoxib treatment (*p* = 0.14).

**TABLE 2:** Simple linear regression results evaluating the association between the duration of firocoxib treatment in the F (firocoxib) group with specific laboratory variables.

Variable	Coefficient	*p*
White blood cells (10^3^cells/uL)	−0.02	0.07
Neutrophils (cells/uL)	−0.02	0.13
Sodium (mmol/L)	0.00	0.73
Potassium (mmol/L)	0.00	0.70
Chloride (mmol/L)	−0.01	0.73
PCV (%)	−0.02	0.70
Manual total protein (g/dL)	0.00	0.99
Creatinine (g/dL)	−0.23	0.97
BUN (g/dL)	0.04	0.21
Albumin (g/dL)	0.00	0.12
Globulin (g/dL)	0.01	0.11

As a post hoc analysis, the subgroup of Arabian horses was analyzed and those receiving firocoxib were compared to those not receiving firocoxib (Table 3). In this post hoc analysis of the Arabian subgroup, the differences in sodium concentration (*p* = 0.02) and total protein concentration (*p* = 0.03) were still present, but there was no longer a difference between white cell counts (*p* = 0.45).

**TABLE 3:** For the subgroup of Arabian horses enrolled in the study, Comparison of laboratory values between the F (firocoxib) and NF (no firocoxib) groups.

Variable	Firocoxib (F)	*N*	No firocoxib (NF)	*N*	*p*	Reference range
White blood cells (WBC; 10^3^ cells/uL)	6.1 (4.3–14.4)	19	6.3 (4.0–10.3)	17	0.45	5.00–12.00
Neutrophils (10^3^ cells/uL)	3.8 (2.4–12.1)	19	3.5 (2.7–7.5)	17	0.30	2.18–6.96
Packed cell volume (PCV; %)	34 (28–40)	19	34 (29–52)	17	0.81	30.0–49.0
Total protein (TP) – manual (g/dL)	6.8 ± 0.4	19	6.5 ± 0.4	17	0.03	5.5–8.0
Globulins (g/dL)	3.8 ± 0.6	19	3.8 ± 0.5	17	0.25	2.8–4.8
Albumin (g/dL)	3.0 ± 0.2	19	3.0 ± 0.2	17	0.95	2.8–3.9
Creatinine (mg/dL)	0.8 (0.5–1.4)	19	0.8 (0.6–0.9)	17	0.92	0.7–1.8
Blood urea nitrogen (BUN; mg/dL)	17.6 ± 4.2	19	18.0 ± 4.3	17	0.77	10.0–25.0
Sodium (Na; mmol/L)	140 (135–142)	19	138 (135–142)	17	0.02	130–142
Potassium (K; mmol/L)	3.8 ± 0.6	19	4.2 ± 0.7	17	0.07	2.4–4.6
Chloride (Cl; mmol/L)	98 ± 5	19	98 ± 4	17	0.46	95–108

*Note:* Data are reported as mean ± SD or median (range).

For horses in the NF group, no history of firocoxib prescriptions from outside pharmacies was present, but records from outside pharmacies were not requested. There were no reports of horses in the NF group receiving firocoxib and then discontinuing due to adverse effects.

## 4 Discussion

This retrospective study compared a group of horses receiving long‐term firocoxib (F) with a group of horses that were not receiving firocoxib (NF). The horses in the firocoxib group were older and were more likely to be of Arabian breeding. A small, but clinically unimportant increase in plasma sodium concentration and total protein concentration was observed in the firocoxib group. There was no difference in BUN and creatinine concentration between the F and NF groups. A small, but clinically unimportant decrease in the total white cell count was observed in the firocoxib group. Duration of firocoxib administration was not associated with changes in any of the variables evaluated in this study.

The age of horses receiving firocoxib (F) in this study was 19 (5–33) years and was significantly higher than 15 (1–33) years of age of horses not receiving firocoxib (NF). This difference could be explained by the high prevalence of lameness in horses over 15 years of age that is associated with osteoarthritis [20]. In the authors' experience, there is also a belief by some clients that this medication is commonly needed in older horses, and therefore owners may ask about the medication more frequently if they have a horse that is aged.

The most common breeds seen in this study were Quarter Horse, Arabian, Thoroughbred, and Paint Horse. Similarly, Quarter Horse and Thoroughbred were the most common breeds in a study evaluating the effects of firocoxib for the management of naturally occurring osteoarthritis [8]. The number of Arabian horses receiving long‐term firocoxib was significantly higher than Arabians not receiving firocoxib. The hospital is located in an area with a large number of endurance horses, and therefore there are a large number of retired Arabian horses being used for trail riding. This may explain the overrepresentation of this breed in the (F) group. In the post hoc analysis of the Arabian subgroup, the differences in sodium concentration and total protein concentration were still present, but there was no longer a difference between white cell counts. However, the size of the group was much smaller for this subset.

One of the unexpected findings in this study was the change in plasma sodium concentration associated with long‐term firocoxib administration. The difference between groups was only 1 mmol/L, which is unlikely to be of clinical significance and would not exceed the normal range for the variable. The highest sodium concentration in the firocoxib group was 143 mmol/L. In human studies, a similar effect was observed where sodium concentration was increased in association with COX‐2 inhibition [19]. The COX‐2 inhibition resulted in a clinically unimportant transient sodium retention without affecting the glomerular filtration rate in healthy, elderly humans [21].

The total protein concentration was minimally increased in horses receiving long‐term firocoxib. In a study evaluating the difference between firocoxib and flunixin meglumine in horses, the total protein was significantly greater after a 5‐day treatment with firocoxib, without significant changes in albumin or globulins [22]. The study hypothesized that this increase in total protein concentration could be caused by an increase in acute phase inflammatory proteins or a change in circulatory volume [22]. In the present study, the use of long‐term firocoxib was not associated with a decrease in total protein, which might be expected given the association between NSAID use in horses and the development of right dorsal colitis [23, 24]. Additional prospective studies could better evaluate whether this change in total protein concentration is repeatable.

The white blood cell count was decreased in horses receiving long‐term firocoxib. In the group receiving firocoxib, age was not associated with white cell count and therefore was unlikely to be a factor in this finding. In general, leukopenia can be found in horses with gastrointestinal disease, and specifically colitis [25, 26]. It is possible that the horses in the present study had subclinical right dorsal colitis associated with NSAID administration; however, the classic hypoproteinemia was not observed. Horses with right dorsal colitis have variable changes in total white cell counts [23], with some horses above and below the reference range in a recent study [24].

While small changes in creatinine concentrations were observed in dogs receiving long‐term firocoxib, there were no significant differences in creatinine and blood urea nitrogen (BUN) in horses receiving firocoxib in this study [3]. A study comparing the use of firocoxib and flunixin meglumine for 5 days in horses found a significant increase in the creatinine concentration during this short treatment period for either treatment [22]. Similarly, when firocoxib was combined with phenylbutazone for 10 days, increases in creatinine were also observed [6]. Overall, the present study suggests that long‐term use of firocoxib for up to 52 months in horses is unlikely to be associated with increases in serum creatinine.

A major limitation of this study was its retrospective nature, which makes it difficult to determine a treatment effect without significant bias. Horses in the study could have been evaluated by another veterinary hospital and received other nephrotoxic drugs, and this information would not be available in the medical records provided. Similarly, it is very difficult to know if owner compliance was acceptable. Clients could have purchased firocoxib for one horse but used the medication for two horses or not given the medication at all. An inherent assumption in this study was that if owners continued to purchase firocoxib, then the horses were receiving firocoxib at the appropriate and prescribed dose.

Another limitation of this study was the difference in ages between the two groups. This was not an unexpected finding because osteoarthritis was one of the most common conditions identified in the study group receiving firocoxib. Osteoarthritis is more common in older horses compared to younger horses [20]. A future prospective study that is randomized and blinded could address this limitation and evaluate whether the results of this study are consistent in age‐matched groups.

Another important limitation of the study design is that a horse that started treatment with firocoxib might have stopped treatment prior to the date of laboratory testing. An example would be an animal that developed loose manure during treatment with firocoxib, and the medication could have been stopped before laboratory testing. The authors are not specifically aware of any horses that fell into this category during this period; however, this bias could result in firocoxib horses being effectively removed from the study if they were having problems with the medication. A prospective study could address this concern.

The cutoff for inclusion in this study was a minimum of 2 months duration of firocoxib treatment. In people, 3 months has been considered a time point for establishing chronic NSAID use [27]. Given that firocoxib is commonly sold to horse clients in multiples of 60 tablets (2, 4, 6 month, etc.), the authors chose 2 months as a cutoff for enrollment in the study. The duration of treatment was not associated with any of the laboratory variables, but the analysis was repeated with a 4‐month minimum treatment duration in order to verify the robustness of the results.

In conclusion, the results of this study suggest that horses receiving long‐term firocoxib at a dose of approximately 0.13 mg/kg orally once a day have minimal changes in biochemical and hematological values. However, oral firocoxib is currently labeled for a 14‐day treatment course.

## Disclosure

Authors declare no off‐label use of antimicrobials.

## Ethics Statement

Authors declare no institutional animal care and use committee or other approval was needed. Authors declare human ethics approval was not needed.

## Conflicts of Interest

The authors declare no conflicts of interest.

## References

[1] M. S. Bergh and S. C. Budsberg , “The Coxib NSAIDs: Potential Clinical and Pharmacologic Importance in Veterinary Medicine,” Journal of Veterinary Internal Medicine 19 (2005): 633–643. 16231707 10.1892/0891-6640(2005)19[633:tcnpca]2.0.co;2

[2] M. Y. Doucet , A. L. Bertone , D. Hendrickson , et al., “Comparison of Efficacy and Safety of Paste Formulations of Firocoxib and Phenylbutazone in Horses With Naturally Occurring Osteoarthritis,” Journal of the American Veterinary Medical Association 232, no. 1 (2008): 91–97. 18167116 10.2460/javma.232.1.91

[3] V. Kvaternick , M. Pollmeier , J. Fischer , et al., “Pharmacokinetics and Metabolism of Orally Administered Firocoxib, a Novel Second Generation Coxib, in Horses,” Journal of Veterinary Pharmacology and Therapeutics 30, no. 3 (2007): 208–217. 17472652 10.1111/j.1365-2885.2007.00840.x

[4] A. Autefage , F. M. Palissier , E. Asimus , and C. Pepin‐Richard , “Long‐Term Efficacy and Safety of Firocoxib in the Treatment of Dogs With Osteoarthritis,” Veterinary Record 168, no. 23 (2011): 617. 21642297 10.1136/vr.d1456

[5] A. Rangel‐Nava , J. M. Ramírez‐Uribe , S. Recillas‐Morales , J. A. Ibancovichi‐Camarillo , A. Venebra‐Muñoz , and P. Sánchez‐Aparicio , “Pharmacological Regulation in the USA and Pharmacokinetics Parameters of Firocoxib, a Highly Selective Cox‐2, by Pain Management in Horses,” Journal of Equine Veterinary Science 77 (2019): 36–42. 31133314 10.1016/j.jevs.2019.02.007

[6] L. Kivett , J. Taintor , and J. Wright , “Evaluation of the Safety of a Combination of Oral Administration of Phenylbutazone and Firocoxib in Horses,” Journal of Veterinary Pharmacology and Therapeutics 37, no. 4 (2014): 413–416. 24354928 10.1111/jvp.12097

[7] O. Lecoindre and C. Pepin‐Richard , “Tolerance of Firocoxib in Dogs With Osteoarthritis During 90 Days,” Journal of Veterinary Pharmacology and Therapeutics 34, no. 2 (2011): 190–192. 21395612 10.1111/j.1365-2885.2010.01227.x

[8] J. A. Orsini , W. G. Ryan , D. S. Carithers , and R. C. Boston , “Evaluation of Oral Administration of Firocoxib for the Management of Musculoskeletal Pain and Lameness Associated With Osteoarthritis in Horses,” American Journal of Veterinary Research 73, no. 5 (2012): 664–671. 22533398 10.2460/ajvr.73.5.664

[9] M. Koene , X. Goupil , C. Kampmann , P. D. Hanson , D. Denton , and M. G. Pollmeier , “Field Trial Validation of the Efficacy and Acceptability of Firocoxib, a Highly Selective Cox‐2 Inhibitor, in a Group of 96 Lame Horses,” Journal of Equine Veterinary Science 30, no. 5 (2010): 237–243.

[10] L. M. Richardson , C. M. Whitfield‐Cargile , N. D. Cohen , A. M. Chamoun‐Emanuelli , and H. J. Dockery , “Effect of Selective Versus Nonselective Cyclooxygenase Inhibitors on Gastric Ulceration Scores and Intestinal Inflammation in Horses,” Veterinary Surgery 47, no. 6 (2018): 784–791. 30094858 10.1111/vsu.12941

[11] N. Hovanessian , J. L. Davis , H. C. McKenzie, 3rd , J. L. Hodgson , D. R. Hodgson , and M. V. Crisman , “Pharmacokinetics and Safety of Firocoxib After Oral Administration of Repeated Consecutive Doses to Neonatal Foals,” Journal of Veterinary Pharmacology and Therapeutics 37, no. 3 (2014): 243–251, 10.1111/jvp.12082. 24749691

[12] A. K. Stuart , B. KuKanich , L. S. Caixeta , J. F. Coetzee , and E. A. Barrell , “Pharmacokinetics and Bioavailability of Oral Firocoxib in Adult, Mixed‐Breed Goats,” Journal of Veterinary Pharmacology and Therapeutics 42, no. 6 (2019): 640–646. 31435966 10.1111/jvp.12795

[13] P. V. Steagall , F. B. Mantovani , T. H. Ferreira , et al., “Evaluation of the Adverse Effects of Oral Firocoxib in Healthy Dogs,” Journal of Veterinary Pharmacology and Therapeutics 30, no. 3 (2007): 218–223. 17472653 10.1111/j.1365-2885.2007.00842.x PMC7194286

[14] K. E. Joubert , “The Effects of Firocoxib (Previcox) in Geriatric Dogs Over a Period of 90 Days,” Journal of the South African Veterinary Association 80, no. 3 (2009): 179–184. 20169752 10.4102/jsava.v80i3.198

[15] M. C. Hochberg , “New Directions in Symptomatic Therapy for Patients With Osteoarthritis and Rheumatoid Arthritis,” Seminars in Arthritis and Rheumatism 32, no. 3 Suppl 1 (2002): 4–14. 12528069 10.1053/sarh.2002.37215

[16] S. P. Curtis , B. Bockow , C. Fisher , et al., “Etoricoxib in the Treatment of Osteoarthritis Over 52‐Weeks: A Double‐Blind, Active‐Comparator Controlled Trial [NCT00242489],” BMC Musculoskeletal Disorders 6 (2005): 58. 16321158 10.1186/1471-2474-6-58 PMC1327669

[17] G. W. Cannon , J. R. Caldwell , P. Holt , et al., “Rofecoxib, a Specific Inhibitor of Cyclooxygenase 2, With Clinical Efficacy Comparable With That of Diclofenac Sodium: Results of a One‐Year, Randomized, Clinical Trial in Patients With Osteoarthritis of the Knee and Hip. Rofecoxib Phase III Protocol 035 Study Group,” Arthritis and Rheumatism 43, no. 5 (2000): 978–987. 10817549 10.1002/1529-0131(200005)43:5<978::AID-ANR4>3.0.CO;2-0

[18] K. A. Brown , E. N. Gregorio , D. Barot , et al., “Single‐Dose Nonsteroidal Anti‐Inflammatory Drugs in Horses Have No Impact on Concentrations of Cytokines or Growth Factors in Autologous Protein Solution and Platelet‐Rich Plasma,” American Journal of Veterinary Research 15 (2024): 1–9. 10.2460/ajvr.23.11.0258 38346393

[19] R. A. Araújo , N. A. A. Sales , R. C. Basile , et al., “Safety Assessment of an Oral Therapeutic Dose of Firocoxib on Healthy Horses,” Veterinary Sciences 10, no. 9 (2023): 531. 37756053 10.3390/vetsci10090531 PMC10535825

[20] P. R. van Weeren and W. Back , “Musculoskeletal Disease in Aged Horses and Its Management,” Veterinary Clinics of North America. Equine Practice 32, no. 2 (2016): 229–247. 27449390 10.1016/j.cveq.2016.04.003

[21] D. C. Brater , C. Harris , J. S. Redfern , and B. J. Gertz , “Renal Effects of COX‐2‐Selective Inhibitors,” American Journal of Nephrology 21, no. 1 (2001): 1–15. 11275626 10.1159/000046212

[22] R. C. Bishop , P. A. Wilkins , A. M. Kemper , R. M. Stewart , and A. M. McCoy , “Effect of Firocoxib and Flunixin Meglumine on Large Colon Mural Thickness of Healthy Horses,” Journal of Equine Veterinary Science 126 (2023): 104562. 37172749 10.1016/j.jevs.2023.104562

[23] J. L. Davis , “Nonsteroidal Anti‐Inflammatory Drug Associated Right Dorsal Colitis in the Horse,” Equine Veterinary Education 29 (2017): 104–113.

[24] J. Flood , D. Byrne , J. Bauquier , et al., “Right Dorsal Colitis in Horses: A Multicenter Retrospective Study of 35 Cases,” Journal of Veterinary Internal Medicine 37, no. 6 (2023): 2535–2543. 37800408 10.1111/jvim.16884 PMC10658563

[25] S. A. Givan , K. E. Estell , J. Martinez‐Lopez , J. A. Brown , D. M. Wong , and S. R. Werre , “Risk Factors Associated With Development of Colitis in Horses Post‐Exploratory Laparotomy,” Equine Veterinary Journal 56, no. 6 (2024): 1162–1169, 10.1111/evj.14028. 37935457

[26] M. C. Roberts , L. L. Clarke , and C. M. Johnson , “Castor‐Oil Induced Diarrhoea in Ponies: A Model for Acute Colitis,” Equine Veterinary Journal 7 (1989): 60–67. 10.1111/j.2042-3306.1989.tb05658.x 9118109

[27] Y. Zhou , D. M. Boudreau , and A. N. Freedman , “Trends in the Use of Aspirin and Nonsteroidal Anti‐Inflammatory Drugs in the General U.S. Population,” Pharmacoepidemiology and Drug Safety 23, no. 1 (2014): 43–50. 23723142 10.1002/pds.3463

